# Are breast cancer patients with suboptimal adherence to cardiovascular treatment more likely to discontinue adjuvant endocrine therapy? Competing risk survival analysis in a nationwide cohort of postmenopausal women

**DOI:** 10.1186/s12916-023-03156-3

**Published:** 2023-11-24

**Authors:** Juliette Artignan, Perrine Capmas, Henri Panjo, Panayotis Constantinou, Nathalie Pelletier-Fleury

**Affiliations:** 1 Centre for Research in Epidemiology and Population Health (Inserm U1018), Villejuif, France; 2 Paris-Saclay University, UVSQ, Inserm, CESP, Villejuif, France; 3 Department of Gynecology and Obstetrics, Kremlin-Bicêtre Hospital, Le Kremlin-Bicêtre, France; 4 Direction of Strategy, Studies and Statistics, French National Health Insurance (CNAM), Paris, France

**Keywords:** Multimorbidity, Breast cancer, Adjuvant endocrine therapy, Cardiovascular risk, Administrative claims data, Adherence

## Abstract

**Background:**

High rates of discontinuation undermine the effectiveness of adjuvant endocrine therapy (AET) among hormone-receptive breast cancer patients. Patient prognosis also relies on the successful management of cardiovascular risk, which affects a high proportion of postmenopausal women. As with AET, adherence with cardiovascular drugs is suboptimal. We examined whether patient adherence with cardiovascular drugs was associated with the rate of AET discontinuation in a French nationwide claims database linked with hospitalisation data.

**Methods:**

We identified postmenopausal women starting AET between 01/01/2016 and 31/12/2020 and taking at least two drugs for the primary prevention of cardiovascular disease (antihypertensive drugs, lipid-lowering drugs and platelet aggregation inhibitors) before AET initiation. Adherence was assessed for each drug class by computing the proportion of days covered. Women were categorised as fully adherent, partially adherent or fully non-adherent with their cardiovascular drug regimen based on whether they adhered with all, part or none of their drugs. AET discontinuation was defined as a 90-day gap in AET availability. Time to AET discontinuation according to levels of cardiovascular drug adherence was estimated using cumulative incidence curves, accounting for the competing risks of death and cancer recurrence. Multivariate cause-specific Cox regressions and Fine-and-Gray regressions were used to assess the relative hazards of AET discontinuation.

**Results:**

In total, 32,075 women fit the inclusion criteria. Women who were fully adherent with their cardiovascular drugs had the lowest cumulative incidence of AET discontinuation at any point over the 5-year follow-up period. At 5 years, 40.2% of fully non-adherent women had discontinued AET compared with 33.5% of partially adherent women and 28.8% of fully adherent women. Both partial adherence and full non-adherence with cardiovascular drugs were predictors of AET discontinuation in the two models (cause-specific hazard ratios 1.16 [95% CI 1.10–1.22] and 1.49 [95% CI 1.39–1.58]; subdistribution hazard ratios 1.15 [95% CI 1.10–1.21] and 1.47 [95% CI 1.38–1.57]).

**Conclusion:**

Clinicians should be aware that patients who do not adhere with their entire cardiovascular drug regimen are also more likely to discontinue AET. This stresses the importance of integrated care, as suboptimal adherence with both treatment components poses a threat to achieving ideal patient outcomes.

**Supplementary Information:**

The online version contains supplementary material available at 10.1186/s12916-023-03156-3.

## Background

Robust evidence has demonstrated the benefits of adjuvant endocrine therapy (AET) on the health outcomes of patients with oestrogen-receptor positive breast cancer (BC). Early trials showed that tamoxifen reduced the rate of BC recurrence by almost 50% in the first 5 years and 30% in the following five. For the first 15 years from treatment initiation, the annual BC death rate was around 30% lower in the treatment groups compared with the control groups [1, 2]. Later trials showed that aromatase inhibitors were more effective than tamoxifen among postmenopausal women [3] and have since been the favoured treatment option in this population. Recent evidence supports the extension of AET duration from 5 to 10 years for additional clinical gains [4, 5] although the best treatment duration remains a debated question [6].

Improvement of cancer treatments have resulted in BC patients being increasingly affected by competing morbidity and mortality risks, one of which is cardiovascular disease (CVD). In women with BC aged over 65, cardiovascular mortality actually exceeds deaths from breast cancer [7]. This can be explained by the shared risk factors for CVD and BC including diet, obesity, sedentary lifestyle but above all age, which increases the probability of developing hypertension and hyperlipidaemia [8]. In France, an estimated 48% of women aged 55 to 64 have hypertension and 25% have hypercholesterolemia. These numbers respectively reach 62 and 31% among women aged 65 to 74 [9, 10]. The strong intersections between cardiovascular risk and breast cancer call for the concurrent management of both conditions to ensure ideal patient outcomes, particularly in postmenopausal women.

Suboptimal medication adherence with AET and cardiovascular medication are well documented and affect patient outcomes. Adherence behaviours are often divided into three components: treatment initiation, implementation and persistence. They indicate whether a patient starts the treatment, respects the dosage schedule and persists with treatment during the intended duration of the prescription. A considerable number of patients discontinue AET before the end of the required 5 years, which negatively impacts disease-free survival [11–13]. Studies report rates of discontinuation on average around 33% [14]. Non-adherence with antihypertensive and lipid-lowering medication is also widespread and is associated with uncontrolled blood pressure, higher incidence of CVD and higher mortality [15].

Much effort has been put into understanding which factors contribute to medication-related behaviours. Nevertheless, the question of overall drug adherence in patients with multiple medications has received little attention. In a sample of Medicare beneficiaries in the USA initiating statins after a hospitalisation for coronary heart disease, patients with low antihypertensive adherence were more likely to discontinue treatment within one year [16]. This behaviour may stretch to chronic conditions outside the cardiovascular sphere. At the time of their BC diagnosis, many women are already at risk of CVD and are treated with multiple antihypertensive and/or lipid-lowering drugs. A meta-analysis on the data of over 375,000 patients showed that patients treated for the primary prevention of CVD were less adherent with these medications than those who had already had a myocardial infarction [17]. Women with low adherence to cardiovascular treatment may be more likely to discontinue AET, placing them doubly at risk of BC mortality and negative CVD-related outcomes. Understanding how women adhere to different components of their treatment is an important step towards helping clinicians best support their patients and encourage physician collaboration in the well-established field of cardio-oncology [18]. The aim of this study was to examine the association between adherence with cardiovascular drug regimens and discontinuation of AET over 5 years, in patients with non-metastatic breast cancer and treated for the primary prevention of CVD.

## Methods

### Database

We used the French National Health Data System (Système National des Données de Santé, SNDS), which merges the national claims database with the national hospital-discharge summaries database and the national death registry through a unique pseudonymised patient identifier. It includes 98.8% of the French population. Individual-level data is collected on all areas of healthcare use. This includes outpatient visits, dispensed medication, procedures, long-term disease (LTD) status, hospital procedures and admission diagnoses using the International Classification of Diseases, 10th revision (ICD-10) codes. A morbidity mapping tool based on algorithms that combine information on LTD status, inpatient diagnoses and pharmacy reimbursement claims allow for the identification of 56 health conditions including CVD and BC. Patient status for each health condition is defined per calendar year. Demographic information such as month and year of birth, date of death and municipality of residence are also recorded [19].

### Population

We identified all women aged over 50 initiating AET between 01/01/2016 and 31/12/2020 with an aromatase inhibitor (anastrozole, letrozole or exemestane). This was defined as the index date. Women were considered to be initiating treatment if they had no reimbursement for any kind of AET in the previous 2 years. We only kept patients who had undergone lumpectomy or mastectomy in the 18 months prior to the index date or in the 6 following months and had no ICD-10 codes for metastasis (C77, C78 and C79 except C773) over the same period.

Women were considered to be under multiple cardiovascular drugs in the year prior to AET initiation if they were reimbursed for at least two antihypertensive and/or lipid-lowering drug classes in the year before that date, with at least 3 reimbursements per drug class. The following therapeutic drug classes were considered: diuretics, beta blocking agents, calcium channel blockers, renin-angiotensin system (RAS) inhibitors (angiotensin-converting enzyme inhibitors [ACEI] or angiotensin-receptor blockers [ARB]), platelet aggregation inhibitors and lipid-modifying agents. Many patients with cardiovascular risk factors are prescribed a multiple drug regimen, which is why we focused on patients with at least two medications. Using the morbidity mapping tool, we excluded women with a CVD diagnosis (acute or chronic coronary syndrome, stroke, acute or chronic heart failure, peripheral arterial disease of the lower limbs) or with end-stage renal disease from the start of the calendar year preceding AET initiation up to the initiation date. Our cohort therefore consisted of women treated for the prevention of CVD. We further excluded women who died within 6 months of AET initiation, did not reside in metropolitan France or for whom information on the French Index of Social Deprivation (FDEP) was missing. The FDEP is a composite measure defined at the municipal level that summarises median household income, percentage of high school graduates in the population aged over 15, percentage of blue-collar workers in the active population and unemployment rate [20].

### Outcome

The primary endpoint was discontinuation of AET, defined as the first day of a period of at least 90 days without any available medication. This is a standard threshold used in the literature [21]. While our population initiated AET with an aromatase inhibitor, we considered supplies of both aromatase inhibitors and tamoxifen during follow-up. Endocrine therapy is supplied in boxes of 28, 30 or 90 pills and dosage is of one pill a day, with a few well-identified exceptions where women are required to take two 10 mg tamoxifen pills a day (see additional Table S1).

The dispensing dates and number of pills supplied were used to create individual 5-year supply diaries starting from the index date and indicating whether a patient had a pill available during each day of follow-up. Patients could stockpile pills for a maximum of 180 days. When a patient refilled their medication before the end of the previous fill, the days of overlap were credited at the end of the next supply. Time-to-discontinuation was measured in days.

### Predictor of interest

The predictor of interest was global cardiovascular treatment adherence in the year before AET initiation. We used a time-invariant measure because it was difficult to make reliable hypotheses about treatment prescriptions over several years without prescription data. Changes in cardiovascular drug regimens over time, such as drug switches and adding or removing therapeutic classes, occur for some patients.

We based patients’ prescriptions off the therapeutic drug classes they were dispensed in the second year before the index date. Supply diaries were created for each drug class in a 1-year look-back period before AET initiation, using the same process as with AET. We additionally considered that patients were supplied their cardiovascular drugs by the hospital staff during hospitalisations requiring an overnight stay. Supplies of all drugs within a given class were considered for creating class-specific diaries. Daily dosage was ruled as one pill a day, which is in line with the usual drug dosage for the primary prevention of CVD.

From the diaries, we estimated patient adherence for each drug class by computing the proportion of days covered (PDC). The numerator was the number of days in the year during which the patient had an available pill of that class, and the denominator was 365. Patients were considered as adherent with a drug class if the corresponding PDC was greater than 80% [22].

We created a 3-level indicator of global cardiovascular treatment adherence in the year before AET initiation by categorising women as follows [23]: fully adherent if they were adherent with all their cardiovascular drug classes, partially adherent if they were adherent with at least one class and not the other(s), and fully non-adherent if they were non-adherent with all their drug classes.

### Statistical analysis

Descriptive statistics were computed at baseline for clinically relevant predictors of AET discontinuation identified in the literature: age, type of BC surgery, adjuvant chemotherapy, number of comorbidities besides BC (list of health conditions available in additional Table S2), number of AET drug switches during follow-up and number of hospitalisations in the year prior to AET initiation [24, 25]. We considered only overnight hospital stays and excluded those that were associated with surgery of the breast. We also included the number of prescribed cardiovascular drugs, an indicator of whether the patient was receiving financial aid for complementary healthcare insurance, and the French index of social deprivation (FDEP). Categorical variables were reported as percentages. We identified a source of potential selection bias arising from the fact that our population did not include patients prescribed multiple antihypertensive/lipid-lowering drugs or platelet aggregation inhibitors in the past but having discontinued treatment by the start of our 2-year look-back period. We therefore planned to adjust for this in our upcoming multivariate models by creating an indicator of time elapsed since cardiovascular treatment initiation. This accounts for the fact that patients with a long-standing treatment are patients that have persisted with treatment for several years and thus may be more adherent.

Patients were followed from the index date until AET discontinuation, 5 years after treatment initiation, December 31st, 2021, death or cancer recurrence, whichever came first. Cancer recurrence was defined as undergoing chemotherapy or radiotherapy, having a second breast surgery or an ICD-10 code for metastasis 6 months or more after the index date.

Standard survival analysis techniques estimate the incidence of the endpoint based on a hypothesis of independent censoring. This implies that censored individuals should not have a higher or lower risk of discontinuing AET, which does not stand when a patient is censored because of death or cancer recurrence. We therefore used a competing risk methodology using cumulative incidence curves to evaluate time-to-discontinuation of AET while accounting for the concurrent risk of death and cancer recurrence. We computed these curves for each level of cardiovascular drug adherence and used Gray’s test to evaluate the hypothesis of equality between the cumulative incidence functions [26]. Univariate and multivariate cause-specific Cox regressions were performed to estimate cause-specific hazard ratios and 95% confidence intervals on all three competing events. We also computed subdistribution hazard ratios using a Fine-and-Gray model to assess the impact of covariates on the cumulative incidence function [27]. The proportional hazards assumption was evaluated by inspecting the log-minus-log plots.

Multivariate models were adjusted using all the variables described in the descriptive statistics. AET drug switches were included as a time-dependent variable. Continuous age was categorised into 5-year groups up to age 80 and categories under 70 were merged as they had almost identical cumulative incidence curves.

To assess the sensitivity of our findings to the chosen outcome definitions, we performed additional analyses by successively rerunning our models with (1) AET discontinuation defined as 60- and 120-day gaps in treatment availability and (2) the adherence threshold for cardiovascular drugs set at a PDC of at least 70 and 90%.

Analyses were performed using SAS Enterprise Guide 8.3.

## Results

### Characteristics of the study population

**Fig. 1:**
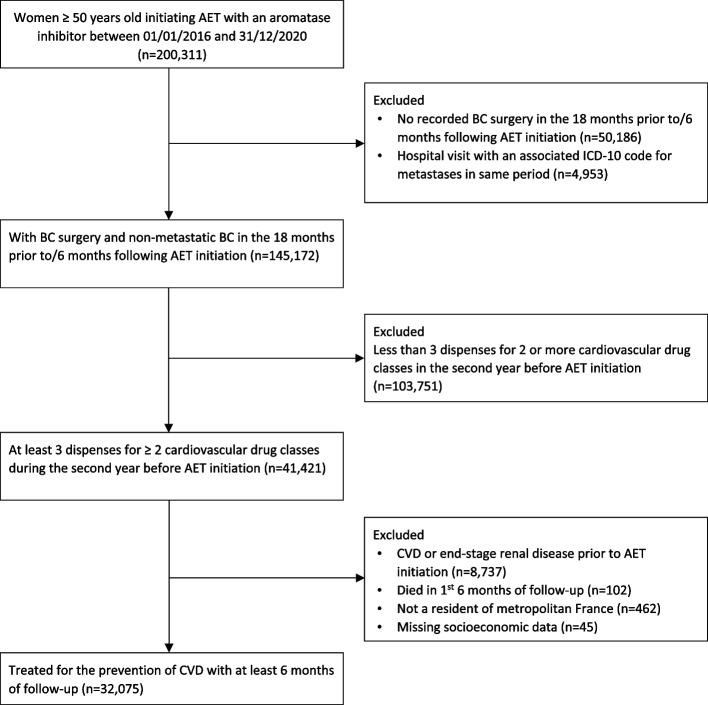
Flowchart of population selection

Details of the population selection process are presented in Fig. 1. Our final cohort consisted of 32,075 women initiating AET for non-metastatic BC and under treatment for the prevention of CVD with a multiple drug regimen for the primary prevention of CVD (median follow-up 29 months [interquartile range: 15–47 months]).

The number of patients supplied with each cardiovascular drug class and proportion of adherent patients by drug class are presented in Table 1. Table 2 presents overall population characteristics (see additional Table S3 for characteristics stratified by level of cardiovascular treatment adherence). The median age was 72 years [IQR 66–78]; 75.7% of women had a lumpectomy and 24.2% a mastectomy; 73.8% had adjuvant chemotherapy. Overall, 43.2% of women were fully adherent with their cardiovascular medication, 43.4% were partially adherent and 13.4% were fully non-adherent.

**Table 1:** Distribution of the Proportion of Days Covered (PDC) according to cardiovascular drug class

Cardiovascular drug class	ATC code	N (total)	Mean	Lower Quartile	Median	Upper Quartile	% of compliant patients^a^
Diuretics	C03	8655	67.8	48.5	83.0	92.9	54.3
Beta blocking agents	C07	14,687	79.8	74.8	89.6	95.6	70.5
Calcium channel blockers	C08	9744	75.5	70.7	89.3	95.6	67.6
Agents acting on the renin–angiotensin system	C09	22,507	83.9	82.5	91.0	95.6	78.9
Lipid-modifying agents	C10	20,917	75.2	67.1	85.8	93.2	61.7
Platelet aggregation inhibitors excl. heparin	B01AC	9226	69.1	57.5	79.5	88.5	49.2

^a^Patients were considered to be adherent with a drug class if their PDC for that class was ≥ 0.8

**Table 2:** Characteristics of the study population

Patient characteristics	% (*n* = 32,075)
**Global cardiovascular adherence**^**a**^	
Full adherence	43.2 (13,855)
Partial adherence	43.4 (13,914)
Full non-adherence	13.4 (4306)
**Age at baseline**	
50–70	44.2 (14,186)
71–75	22.8 (7299)
76–80	15.4 (4945)
> 80	17.6 (5645)
**Type of BC surgery**	
Tumorectomy	76.0 (24,363)
Total mastectomy	24.0 (7712)
**Adjuvant chemotherapy in the year before AET**	
No	74.0 (23,740)
Yes	26.0 (8335)
**Number of prescribed cardiovascular drugs**	
2	37.3 (11,966)
3	37.0 (11,854)
4	25.6 (8255)
5–6	
**Time since initiation of any CV treatment**	52.7 (16910)
< 5 years	31.3 (10,038)
≥ 5 years	12.3 (3958)
**Number of comorbidities (besides BC and CV risk)**	3.6 (1169)
0	
1	13.3 (4269)
≥ 2	86.7 (27,806)
**Number of hospital stays in the year before AET**	
0	80.1 (25,694)
1	14.4 (4611)
≥ 2	5.5 (1770)
**FDEP**^**b**^	
Q1	17.8 (5725)
Q2	18.4 (5894)
Q3	19.6 (6290)
Q4	21.2 (6801)
Q5	23.0 (7365)
**Financial aid for complementary health insurance**	
No	96.3 (30,877)
Yes	3.7 (1198)

Abbreviations: *AET* adjuvant endocrine therapy, *BC* breast cancer, *CV* cardiovascular

^a^Patients were classified as fully adherent, partially adherent or fully non-adherent if they adhered to all, some or none of their cardiovascular drugs

^b^The FDEP is a French index of social deprivation that summarises median household income, percentage of high school graduates in the population aged over 15, percentage of blue-collar workers in the labour force and unemployment rate. Q1 is the least deprived quintile

### Cumulative incidence of AET discontinuation

**Fig. 2:**
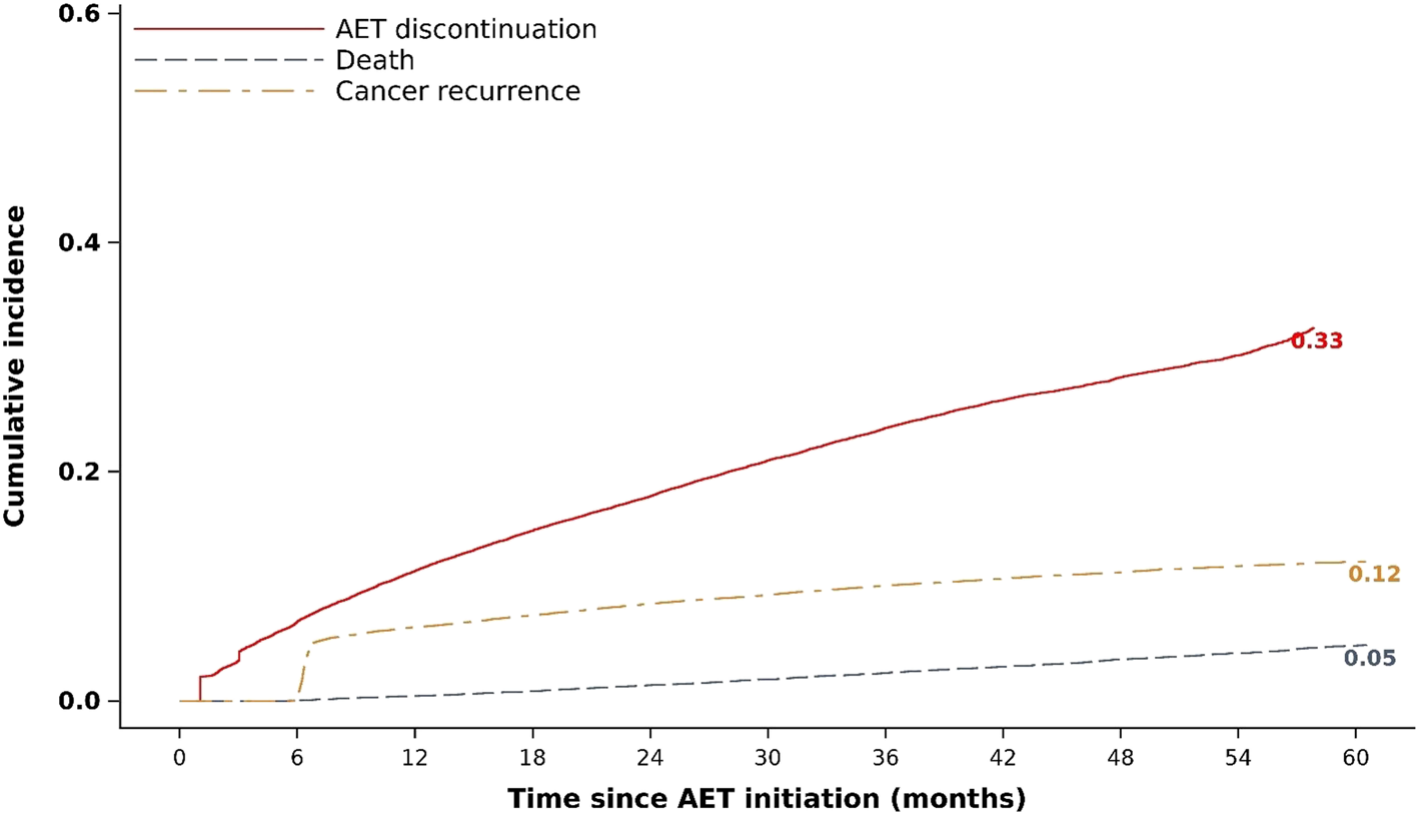
Cumulative incidence of AET discontinuation, death and cancer recurrence over 5 years

**Fig. 3:**
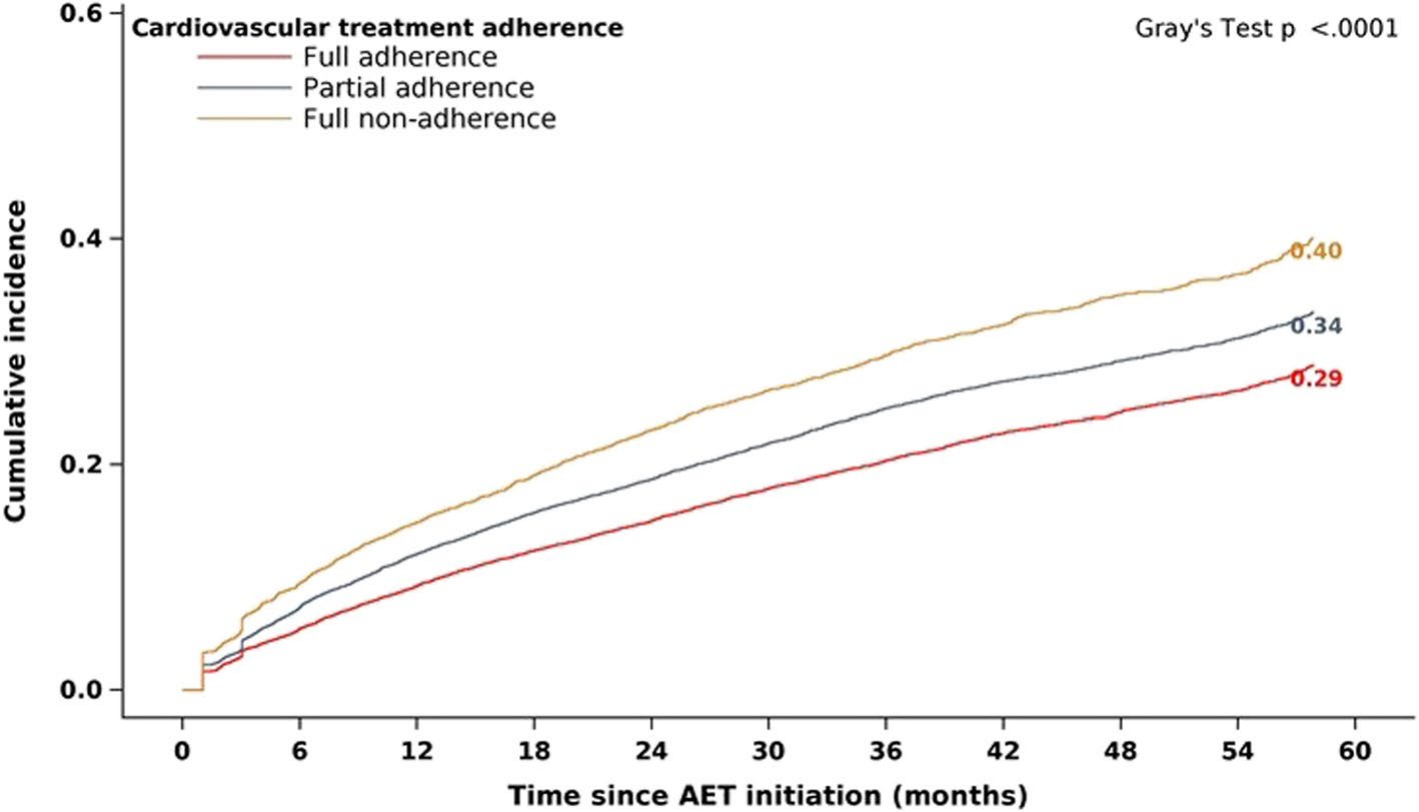
Cumulative incidence of AET discontinuation according to different levels of cardiovascular treatment adherence

By the end of the first year of treatment, 11.3% of the cohort had discontinued AET. Discontinuation reached 17.7% by the end of the second year, 23.8% by the end of the third year, 28.0% by the end of the fourth year and 32.4% at 5 years (Fig. 2). The cumulative incidence of death, that is death that occurred before treatment discontinuation or cancer recurrence, was 4.9% at 5 years, and that of cancer recurrence was 12.6%. At each time point over the 5-year treatment period, the cumulative incidence of AET discontinuation was highest in patients who were fully non-adherent with CV treatment and lowest in those who were fully adherent (Fig. 3). At 5 years, 40.2% of fully non-adherent patients had discontinued AET compared with 33.5% of partially adherent patients and 28.8% of fully adherent patients.

### Predictors of AET discontinuation

Log-minus-log plots for each predictor and unadjusted estimates are available in additional Figure S3 and additional Table S4. Adjusted cause-specific hazard ratios (CSHR) for predictors of AET discontinuation and competing events are presented in Table 3 alongside subdistribution hazard ratios (SDHR) for the Fine-and-Gray regression of AET discontinuation. CSHRs are interpreted as the adjusted effect of each covariate on the instantaneous rate of AET discontinuation among women still event-free, that is, who have not discontinued AET, died or experienced cancer recurrence up to time *t*. Lower levels of cardiovascular medication adherence were associated with an increase in the rate of AET discontinuation. Partially adherent patients had a rate of AET discontinuation 16% higher than those fully adherent (CSHR 1.16 [95% CI 1.10–1.22]) and this increase reached 49% among patients who were fully non-adherent (CSHR 1.49 [95% CI 1.39–1.58]). The rate of occurrence of the competing events was not impacted by cardiovascular adherence. Being partially adherent rather than fully adherent did not significantly change the rate of death (CSHR 0.96 [95% CI 0.84; 1.11]) or cancer recurrence (CSHR 1.05 [95% CI 0.97; 1.13]). Being fully non-adherent did not change the rate of recurrence (CSHR 0.98 [95% CI 0.88; 1.08]) and non-significantly increased the rate of death (CSHR 1.17 [95% CI 0.96; 1.42]).

**Table 3:** Hazard ratios from cause-specific and subdistribution hazard models for predictors of AET discontinuation, death and cancer recurrence

**Predictors (adjusted)**	Cause-specific Cox model			Fine-and-Gray subdistribution model
	AET discontinuation	Death	Cancer recurrence	AET discontinuation
	CSHR^a^ [95% CI]	CSHR [95% CI]	CSHR [95% CI]	SDHR^b^ [95% CI]
**Global cardiovascular adherence**^**c**^				
Total adherence	Ref	Ref	Ref	Ref
Partial adherence	1.16 [1.10; 1.22]***	0.96 [0.84; 1.11]	1.05 [0.97; 1.13]	1.15 [1.10; 1.21]***
Total non-adherence	1.49 [1.39; 1.58]***	1.17 [0.96; 1.42]	0.98 [0.88; 1.08]	1.47 [1.38; 1.57]***
**Age at baseline**				
50–70	Ref	Ref	Ref	Ref
71–75	1.20 [1.13; 1.27] ***	1.69 [1.37; 2.09]***	1.02 [0.93; 1.12]	1.18 [1.11; 1.25]***
76–80	1.44 [1.35; 1.53] ***	2.31 [1.86; 2.86]***	1.38 [1.24; 1.52]***	1.39 [1.30; 1.48]***
> 80	1.90 [1.79; 2.02] ***	6.60 [5.52; 7.90]***	1.78 [1.60; 1.98]***	1.77 [1.66; 1.88]***
**Type of BC surgery**				
Tumorectomy	Ref	Ref	Ref	Ref
Total mastectomy	1.03 [0.98; 1.08]	1.51 [1.32; 1.72]***	1.21 [1.12; 1.30]***	1.01 [0.96; 1.06]
**Adjuvant chemotherapy in the year before AET**				
No	Ref	Ref	Ref	Ref
Yes	0.92 [0.87; 0.98] **	0.80 [0.66; 0.98]**	5.58 [5.17; 6.03]***	0.80 [0.75; 0.84]***
**Number of prescribed cardiovascular drug classes**				
2	Ref	Ref	Ref	Ref
3	1.04 [0.99; 1.09]	1.26 [1.09; 1.46]***	0.96 [0.89; 1.04]	1.04 [0.99; 1.09]
4	1.07 [1.00; 1.15]*	1.39 [1.16; 1.68]***	0.93 [0.83; 1.04]	1.07 [0.99; 1.14]
5–6	1.13 [1.01; 1.27]*	1.57 [1.19; 2.06]***	0.99 [0.82; 1.19]	1.12 [1.00; 1.26]*
**Time since initiation of any cardiovascular treatment**				
< 5 years	Ref	Ref	Ref	Ref
≥ 5 years	0.58 [0.54; 0.61] ***	1.17 [0.95; 1.43]	1.00 [0.91; 1.11]	0.57 [0.54; 0.61]***
**AET drug switch**				
0	Ref	Ref	Ref	Ref
1	1.79 [1.69; 1.91]***	0.98 [0.81; 1.18]	0.81 [0.72; 0.92]***	1.88 [1.76; 2.00]***
≥ 2	2.57 [2.37; 2.78]***	0.65 [0.47; 0.89]**	1.15 [0.97; 1.36]	2.75 [2.53; 2.99]***
**Number of comorbidities (besides BC and CV risk)**				
0	Ref	Ref	Ref	Ref
1	1.17 [1.11; 1.23]***	1.43 [1.20; 1.69]***	0.99 [0.91; 1.07]	1.16 [1.10; 1.22]***
≥ 2	1.23 [1.16; 1.30]***	2.66 [2.26; 3.14]***	1.11 [1.02; 1.21]*	1.20 [1.13; 1.27]***
**Number of hospital stays in the year before AET**				
0	Ref	Ref	Ref	Ref
1	1.05 [0.98; 1.11]	1.30 [1.10; 1.53]**	1.14 [1.04; 1.24]**	1.03 [0.97; 1.10]
≥ 2	1.12 [1.02; 1.23]*	1.69 [1.35; 2.12]***	1.20 [1.07; 1.36]*	1.09 [1.00; 1.20]
**FDEP**^**d**^				
Q1	1.13 [1.06; 1.21]***	0.85 [0.69; 1.04]	0.90 [0.81; 1.01]	1.14 [1.06; 1.22]***
Q2	1.10 [1.03; 1.18]**	0.84 [0.69; 1.02]	0.99 [0.89; 1.09]	1.11 [1.04; 1.19]**
Q3	1.07 [1.00; 1.15]*	0.97 [0.80; 1.16]	0.90 [0.81; 1.00]*	1.08 [1.01; 1.16]*
Q4	1.04 [0.97; 1.11]	0.92 [0.77; 1.10]	0.90 [0.81; 1.00]*	1.05 [0.98; 1.12]
Q5	Ref	Ref	Ref	Ref
**Financial aid for complementary health insurance**				
No	Ref	Ref	Ref	Ref
Yes	1.07 [0.95; 1.20]	1.48 [1.08; 2.01]**	0.97 [0.81; 1.16]	1.07 [0.95; 1.20]

Abbreviations: *BC* breast cancer, *AET* adjuvant endocrine therapy, *CSHR* cause-specific hazard ratio, *SDHR* subdistribution hazard ratio

^a^Cause-specific hazard ratios are interpreted as the adjusted effect of the covariate on the instantaneous rate of AET discontinuation among women currently event-free, that is who have not discontinued AET, died or experienced cancer recurrence up to time t

^b^Subdistribution hazard ratios are interpreted as the impact of a given covariate on the cumulative incidence of AET, that is on the probability that AET discontinuation occurred in the population by time t

^c^Patients were classified as fully adherence, partially adherent or fully non-adherent if they adhered to all, some or none of their cardiovascular drugs

^d^The FDEP is a French index of social deprivation that summarises median household income, percentage of high school graduates in the population aged over 15, percentage of blue-collar workers in the active population and unemployment rate. Q1 is the least deprived quintile

*p ≤ 0.05

**p ≤ 0.01

***p ≤ 0.001

SDHRs describe the impact of a given covariate on the cumulative incidence function, in other words on the cumulative probability that AET discontinuation occurred by time *t*. The Fine-and-Gray model therefore considers how a covariate is related to the competing events, since the value of the SDHRs may result from the direct effect of the covariate on the rate of AET discontinuation or from its indirect effect on the rate of death or cancer recurrence [28]. The Fine-and-Gray model produced SDHRs that were almost identical to the CSHRs with regard to the effect of cardiovascular treatment adherence on AET discontinuation (SDHRs 1.15 [95% CI 1.10; 1.21] and 1.47 [95% CI 1.38; 1.57] for partial adherence and full non-adherence**).**

Switching to a different AET drug was the strongest predictor of treatment discontinuation; the effect of a second switch was even stronger (CSHR 2.57 [95% CI 2.37; 2.78]) than that of a first (CSHR 1.79 [95%: 1.69; 1.91]). Age was also a strong predictor of AET discontinuation. Being in an older age group rather than below 70 progressively increased the rate of discontinuation; the relative hazard was highest for women over 80 (CSHR 1.90 [95% CI 1.79; 2.02]). Women over 80 had a much higher hazard of death (CSHR 6.60 [95% CI 5.52; 7.90]), which explains why being in the oldest age group had a lower impact on the cumulative incidence of AET discontinuation (SDHR 1.77 [95% CI 1.66; 1.88]). Having two or more chronic conditions besides breast cancer and cardiovascular risk was associated with a higher rate of discontinuation (CSHR 1.23 [95% CI 1.16–1.30]) and a higher cumulative incidence of the event (SDHR 1.20 [95% CI 1.13; 1.27]). Adjuvant chemotherapy before starting AET had a slightly negative effect on the rate of treatment discontinuation (CSHR 0.92 [95% CI 0.87; 0.98]) but was associated with a high rate of cancer recurrence (CSHR 5.58 [95% CI 5.17; 6.03]) and had an important impact on the cumulative incidence of AET discontinuation (SDHR: 0.80 [95% CI 0.75; 0.84]). Women who had been taking cardiovascular treatment for over 5 years had a much lower rate of discontinuation (CSHR 0.58 [95% CI 0.54; 0.61]). The impact on the cumulative incidence was identical.

### Sensitivity analysis

These main results were supported by the several sensitivity analyses that we conducted. Lowering the threshold for drug adherence from 80 to 70% increased the size of the main effect, as did lowering the definition of discontinuation from a 90- to a 60-day gap in treatment availability. Increasing these thresholds tended to lower the size of the effect but did not change any qualitative conclusions. Details are available Table 4.

**Table 4:** Results of the sensitivity analysis with varying definitions of cardiovascular drug adherence and AET discontinuation

**Predictors of AET discontinuation (adjusted)**	Cause-specific Cox regression	Fine-and-Gray subdistribution model
	*CSHR [95% CI]*	*SDHR [95% CI]*
***Varying PDC thresholds for adherence***		
**Global cardiovascular adherence (PDC ≥ 0.7)**		
Full adherence	Ref	Ref
Partial adherence	1.16 [1.11; 1.22] ***	1.16 [1.10; 1.21] ***
Full non-adherence	1.58 [1.46; 1.71] ***	1.57 [1.44; 1.70] ***
**Global cardiovascular adherence (PDC ≥ 0.9)**		
Full adherence	Ref	Ref
Partial adherence	1.12 [1.05; 1.20]***	1.12 [1.05; 1.19]***
Full non-adherence	1.37 [1.29; 1.45]***	1.36 [1.28; 1.45]***
***Discontinuation* = *at least 60 days without any available AET pill***		
**Global cardiovascular adherence (pdc ≥ 0.8)**		
Full adherence	Ref	Ref
Partial adherence	1.15 [1.10; 1.20]***	1.14 [1.09; 1.20]***
Full non-adherence	1.53 [1.45; 1.62]***	1.52 [1.43; 1.61]***
***Discontinuation* = *at least 120 days without any available AET pill***		
**Global cardiovascular adherence (PDC ≥ 0.8)**		
Full adherence	Ref	Ref
Partial adherence	1.14 [1.09; 1.21]***	1.14 [1.08; 1.20]***
Full non-adherence	1.44 [1.35; 1.54]***	1.43 [1.33; 1.53]***

Abbreviations: *AET* adjuvant endocrine therapy, *CSHR* cause-specific hazard ratio, *SDHR* subdistribution hazard ratio, *PDC* proportion of days covered

***p ≤ 0.001

## Discussion

We used pharmacy claims and hospitalisation data covering 98.8% of the French population to examine whether adherence with cardiovascular drugs was associated with AET discontinuation in women with non-metastatic oestrogen-receptor positive BC and with a multiple drug regimen for the primary prevention of CVD. At each point over the 5-year period, women who were not adherent with any of their CV drugs in the year prior to AET initiation had a higher cumulative incidence of AET discontinuation. Cause-specific hazard ratios showed that CV adherence was a strong predictor of AET discontinuation when adjusting for frequently associated factors. We observed an increasing gradient between this variable and AET discontinuation. Fully non-adherent women had a 49% higher rate of AET discontinuation over the 5-year follow-up period compared with fully adherent women, and partially adherent women a 16% higher rate of discontinuation. The subdistribution hazard ratios yielded by the Fine-and-Gray model showed an almost identical impact of our exposure of interest on the cumulative incidence of AET discontinuation. This is because cardiovascular treatment adherence was not associated with the rate of occurrence of the competing events. Our results were supported by the sensitivity analyses in which we tested more or less lenient definitions of AET discontinuation (minimum 120- and 60-day gaps in treatment) and of the adherence threshold for the proportion of days covered (70 and 90%). We can therefore consider that our conclusions were not biased by our choice of outcome definitions.

In our study, 17% of the population either died or had cancer recurrence before discontinuing AET. Competing risks are largely prevalent in studies that include older adults with multimorbidity. Failure to account for them correctly can lead to adverse consequences, including overestimating the cumulative incidence of the event of interest and false interpretations of the hazard ratios [29, 30]. These issues have been raised by several authors in the geriatric literature [31, 32] but have rarely been considered when studying the determinants of AET discontinuation. While here cause-specific and subdistribution models yielded almost identical results with regard to our exposure of interest, the effect of some covariates varied more substantially. This vouches for computing both types of models for an accurate description of effect estimates when studying AET discontinuation.

While the number of prescribed medications and of coexisting health conditions are commonly included in models aiming to understand which patients are more likely to discontinue AET, studies have not focused on patients with multimorbidity and their adherence to other medication classes. Our finding of an association between low adherence with cardiovascular treatment and increased hazard of AET discontinuation is consistent with previous studies demonstrating consistent behaviours across different drug classes, mainly between statins and antihypertensive drugs, antidiabetics and other lipid-lowering drugs [16, 33–35]. Our study builds on past research in demonstrating that the association stands when considering very separate disease entities. Its longitudinal aspect shows the association is maintained over the whole 5-year course of AET. It must be mentioned that in France, AET and cardiovascular drugs are available to patients free of charge. Drug class-specific adherence behaviours are likely to be influenced by drug price and when patients bear the cost of medication themselves [36, 37]. Our results should be extrapolated with caution in countries where this is the case.

Understanding global patient adherence to prescribed medication is all the more relevant given the high prevalence of multimorbidity in breast cancer patients. There is currently no standard definition of multimorbidity which makes it challenging to provide accurate prevalence estimates. Nevertheless, studies are in agreement to say that over 50% of patients with BC have at least one co-occurring chronic condition [38, 39]. Hypertension and CVD are high on the list of comorbidities. Not only are a large proportion of women are already at risk of CVD at the time of their BC diagnosis, commonly used anticancer therapies can affect cardiac function and further increase the risk of developing post-cancer heart failure, arterial thromboembolism or myocardial ischaemia and infarction [7, 40]. Other authors suggest women may also be at heightened risk of CVD after a cancer diagnosis due to lifestyle modifications such as deteriorated diet or decreased physical activity over the course of the disease [8, 41]. As a result, the risk of mortality attributable to CVD among postmenopausal women is higher in BC survivors than in women without a history of BC [42].

Non-adherence with preventive medication is a major driver of multimorbidity, and our results show consistent adherence behaviours across cardiovascular treatment and AET. A step towards limiting the burden of multimorbidity in BC cancer patients will therefore require identifying and supporting women with low adherence to their cardiovascular drug regimen early on in the course of AET, as they may be at heightened risk of both CVD and BC-related events. This is an additional argument in favour of “cluster medicine”, a term coined by a BMJ editorial in 2020 to shift the consideration of multimorbidity from a “random assortment of individual conditions to recognising it as a series of largely predictable clusters of disease in the same person” [43] and encourage collaborative care. Helping patients better cope with their treatment may include switching to a less complex cardiovascular drug regimen if feasible, as these tend to facilitate adherence [44], or enrolment in disease self-management programmes [45, 46]. In any case, these solutions should involve different specialists without being focused on a single treatment category. While our results should encourage clinicians to keep in mind that patients with suboptimal past cardiovascular drug adherence are more at risk than others of discontinuing AET, they cannot be used to determine individual risk. Further research could look into building prediction models to find the combination of factors that best predict AET discontinuation and to understand how past adherence fits within these combinations.

There are limitations to our study, mainly linked to our use of an administrative claims database. We used drug refill dates and number of pills supplied to estimate discontinuation and adherence behaviours. While this provides a good indication of whether patients comply with treatment, it gives no indication of whether supplied pills have indeed been ingested. There is currently no gold standard for measuring medication adherence, and claims data are a reliable and non-invasive data source that provide insights into patient behaviour among large community samples [22]. An additional limitation is the lack of prescription data in the SNDS. We hypothesised that patients were under a one daily intake dosing regimen for each antihypertensive and/or lipid-lowering drug that they were supplied. This reflects usual drug dosage for patients treated for the primary prevention of CVD but may have resulted in some degree of inaccuracy. More specifically, class PDCs were underestimated for patients taking half a pill a day, who were mistakenly perceived as picking up too little medication, and overestimated for patients taking several pills a day. Some patients may therefore have been classified in the wrong global cardiovascular adherence category. To our knowledge, women who discontinued AET and those who persisted had an equal risk of being misclassified. This case of non-differential misclassification would tend to reduce the size of our main effect, though we believe this should be limited due to the small proportion of women concerned. This is supported by the fact that the mean PDCs per drug class were in agreement with the usual ranges found in the literature [17], our patients being neither overly adherent nor non-adherent. Another limitation is that patients who had discontinued cardiovascular treatment by the start of our 2-year look-back period were not included in our population, since we selected women who had at least 3 dispenses per medication class in the second year prior to AET initiation. This is also the case for patients who were only reimbursed once or twice during that year. These non-adherent patients may have had a high incidence of AET discontinuation. Although we at least partially adjusted for this by including an indicator of time since cardiovascular treatment initiation in our models, our estimate may be biased towards the null. Finally, the administrative database used in this study did not provide clinical information such as the stage of cancer at diagnosis or histological data, which may affect how women persist with AET. Nevertheless, excluding women with metastatic cancer ensured a certain degree of homogeneity by excluding patients diagnosed at advanced stages. The type of surgical treatment (partial or total mastectomy) also provided information on the stage at diagnosis and slightly nuances this limitation.

## Conclusion

Consequences of high blood pressure and hyperlipidaemia are a leading cause of premature death worldwide, and many affected patients are treated with a multi-pill regimen. One of the leading causes of uncontrolled blood pressure and CVD is non-adherence, and postmenopausal BC patients are especially vulnerable with regard to cardiovascular health. Understanding how multimorbid patients with BC behave towards their entire treatment contributes to identifying patients that may be concurrently at increased risk of negative outcomes for different diseases. Our study suggests that specialist clinicians should pay particular attention to patients if they notice low adherence to a particular drug. This may in fact reveal suboptimal adherence across other medication classes.

### Supplementary Information

**Additional file 1: Tables S1.** List of AET drugs with a dosing regimen of 2 pills per day. **Table S2.** List of health conditions considered for the comorbidity count included in multivariate models. **Table S3.** Population characteristics stratified by level of cardiovascular treatment adherence. **Table S4.** Unadjusted predictors of AET discontinuation using Cox and Fine-and-Gray regressions. **Figures S1.** Log-minus-log plots for each time-invariant predictor.

## Data Availability

Access to the SNDS database was delivered by the National Health Insurance Fund (CNAM, Caisse Nationale d’Assurance Maladie) following approval of the study protocol by the CEPIDC (Centre d’Epidémiologie sur les Causes Médicales de Décès) and the corresponding author undertaking a compulsory training course. Access to the database or a sample of the data is conditional upon participation in a training course and validation of the study protocol by the competent authorities according to the researcher’s institution.

## Abbreviations

AET: Adjuvant endocrine therapy
BC: Breast cancer
CV: Cardiovascular
CVD: Cardiovascular disease
