# Supplementary material for: Are breast cancer patients with suboptimal adherence to cardiovascular treatment more likely to discontinue adjuvant endocrine therapy? Competing risk survival analysis in a nationwide cohort of postmenopausal women
Source: BMC Med. 2023 Nov 24;21:463. doi: 10.1186/s12916-023-03156-3 (PMC10675896; doi:10.1186/s12916-023-03156-3)

Tables S1. List of AET drugs with a dosing regimen of two pills per day

| Drug | CIP-13 drug code^a^ | Dose | Number of pills per pack | Pills per day | Days supplied |
| --- | --- | --- | --- | --- | --- |
| TAMOXIFEN | 3400933263928 | 10 mg | 30 | 2 | 15 |
| TAMOXIFEN | 3400932059324 | 10 mg | 30 | 2 | 15 |
| TAMOXIFEN | 3400932903696 | 10 mg | 30 | 2 | 15 |
| TAMOXIFEN | 3400932921508 | 10 mg | 30 | 2 | 15 |
| TAMOXIFEN | 3400933179441 | 10 mg | 30 | 2 | 15 |
| TAMOXIFEN | 3400933952235 | 10 mg | 30 | 2 | 15 |
| TAMOXIFEN | 3400934106323 | 10 mg | 30 | 2 | 15 |
| TAMOXIFEN | 3400934303623 | 10 mg | 30 | 2 | 15 |
| TAMOXIFEN | 3400934639302 | 10 mg | 30 | 2 | 15 |
| TAMOXIFEN | 3400935646484 | 10 mg | 30 | 2 | 15 |
| TAMOXIFEN | 3400935825407 | 10 mg | 30 | 2 | 15 |
| TAMOXIFEN | 3400935940230 | 10 mg | 30 | 2 | 15 |
| TAMOXIFEN | 3400936143463 | 10 mg | 30 | 2 | 15 |
| TAMOXIFEN | 3400936136601 | 10 mg | 30 | 2 | 15 |
| TAMOXIFEN | 3400936476189 | 10 mg | 30 | 2 | 15 |
| TAMOXIFEN | 3400936797598 | 10 mg | 30 | 2 | 15 |
| TAMOXIFEN | 3400936663954 | 10 mg | 30 | 2 | 15 |
| TAMOXIFEN | 3400933179502 | 10 mg | 30 | 2 | 15 |

^a^ CIP-13 codes are numeric identifiers for pharmacy products

Table S2. List of health conditions considered for the comorbidity count included in the multivariate models

| **Health conditions** |
| --- |
| Diabetes |
| Colorectal cancer |
| Lung cancer |
| Other cancers |
| Psychotic disorders |
| Neurotic disorders |
| Manic and bipolar disorders |
| Depression and other mood disorders |
| Neurotic disorders linked to stress and somatoform disorders |
| Mental disability |
| Alcohol addiction |
| Tabacco addiction |
| Addiction disorders linked to cannabis consumption |
| Other addiction disorders |
| Psychiatric disorders having started in childhood |
| Other psychiatric disorders |
| Alzheimer’s disease |
| Others forms of dementia |
| Parkinson’s disease |
| Multiple sclerosis |
| Paraplegia |
| Myopathy or myasthenia |
| Epilepsy |
| Other neurological conditions |
| Chronic respiratory diseases (except cystic fibrosis) |
| [Rheumatoid arthritis](https://www.linguee.fr/anglais-francais/traduction/rheumatoid+arthritis.html) and associated conditions |
| [Ankylosing spondylitis](https://www.linguee.fr/anglais-francais/traduction/ankylosing+spondylitis.html)  and associated conditions |
| Other chronic inflammatory diseases |
| Hereditary metabolic disease or amylose |
| Cystic fibrosis |
| Hemophilia |
| Other severe hemostasis disorders |
| Rare diseases |
| HIV or AIDS |
| Liver diseases (exluding cystic fibrosis) |
| Pancreas diseases (excluding cystic fibrosis) |
| Chronic hepatitis C |
| Other unfound long-term diseases |
| Long-term disease status for bone marrow impairment and other chronic cytopenias |
| Long-term disease status for complicated bilharziasis |
| Other long-term disease status for Autres affections de longue durée pour hemoglobinopathies, chronic constitutional or acquired hemolysis |
| Other long-term disease status for chronic severe nephropathy and primitive nephrotic syndrome (excluding end-stage renal disease) |
| Other long-term disease status for psychiatric disorders (chromosomic anomalies) |
| Other long-term disease status for progressive structural scoliosis |
| Other long-term disease status for active tuberculosis, leprosy |
| Other long-term disease status for tumors of unknown or unpredictable evolution |
| Other long-term disease status |
| Trisomy 21 |
| Obesity |

Table S3. Population characteristics stratified by level of cardiovascular treatment adherence

|  | **Full adherence** | **Partial adherence** | **Full non-adherence** |
| --- | --- | --- | --- |
|  | **% (n)** | **% (n)** | **% (n)** |
| **Number of cardiovascular drugs** |  |  |  |
| 2 | 63.5 (8797) | 38.0 (5283) | 65.7 (2828) |
| 3 | 26.5 (3667) | 38.1 (5306) | 24.7 (1065) |
| 4 | 8.1 (1126) | 17.9 (2487) | 8.0 (345) |
| 5-6 | 1.9 (264) | 6.0 (837) | 1.6 (67) |
| Total | 100.0 (13854) | 100.0 (13913) | 100.0 (4305) |
| **Age at initiation** |  |  |  |
| ≤ 70 | 46.6 (6462) | 40.9 (5688) | 47.3 (2035) |
| 71-75 | 23.1 (3198) | 23.2 (3230) | 20.2 (870) |
| 76-80 | 14.5 (2011) | 16.6 (2314) | 14.4 (620) |
| > 80 | 15.8 (2183) | 19.3 (2681) | 18.1 (780) |
| Total | 100.0 (13854) | 100.0 (13913) | 100.0 (4305) |
| **Type of BC surgery** |  |  |  |
| Tumorectomy | 76.7 (10626) | 75.6 (10514) | 74.8 (3221) |
| Total mastectomy | 23.3 (3228) | 24.4 (3399) | 25.2 (1084) |
| Total | 100.0 (13854) | 100.0 (13913) | 100.0 (4305) |
| **Time since initiation of any CV treatment** |  |  |  |
| < 5 years | 12.2 (1695) | 13.3 (1851) | 16.8 (723) |
| ≥ 5 years | 87.8 (12159) | 86.7 (12062) | 83.2 (3582) |
| Total | 100.0 (13854) | 100.0 (13913) | 100.0 (4305) |
| **Number of comorbidities (besides BC)** |  |  |  |
| 0 | 38.6 (5354) | 35.5 (4937) | 38.5 (1658) |
| 1 | 36.7 (5079) | 38.0 (5292) | 35.8 (1542) |
| ≥ 2 | 24.7 (3421) | 26.5 (3684) | 25.7 (1105) |
| Total | 100.0 (13854) | 100.0 (13913) | 100.0 (4305) |
| **Number of hospital stays in the year before AET** |  |  |  |
| 0 | 83.2 (11531) | 78.9 (10971) | 74.1 (3189) |
| 1 | 12.8 (1780) | 14.9 (2078) | 17.5 (753) |
| ≥ 2 | 3.9 (543) | 6.2 (864) | 8.4 (363) |
| Total | 100.0 (13854) | 100.0 (13913) | 100.0 (4305) |
| **Chemotherapy in the year before AET** |  |  |  |
| No | 76.3 (10574) | 73.7 (10252) | 67.6 (2911) |
| Yes | 23.7 (3280) | 26.3 (3661) | 32.4 (1394) |
| Total | 100.0 (13854) | 100.0 (13913) | 100.0 (4305) |
| **FDEP^a^** |  |  |  |
| Q1 | 15.8 (2189) | 18.9 (2631) | 21.0 (905) |
| Q2 | 18.4 (2547) | 18.2 (2538) | 18.8 (808) |
| Q3 | 19.7 (2723) | 19.8 (2758) | 18.8 (809) |
| Q4 | 22.0 (3041) | 20.8 (2890) | 20.2 (869) |
| Q5 | 24.2 (3354) | 22.3 (3096) | 21.2 (914) |
| Total | 100.0 (13854) | 100.0 (13913) | 100.0 (4305) |
| **Financial aid for complementary health insurance** |  |  |  |
| No | 96.4 (13361) | 96.5 (13429) | 94.9 (4086) |
| Yes | 3.6 (493) | 3.5 (484) | 5.1 (219) |
| Total | 100.0 (13854) | 100.0 (13913) | 100.0 (4305) |

Abbreviations: *BC* breast cancer; *AET* adjuvant endocrine therapy

^a^ The FDEP is a French index of social deprivation that summarises median household income, percentage of high school graduates in the population aged over 15, percentage of blue-collar workers in the labour force and unemployment rate. Q1 is the least deprived quintile.

Table S4. Unadjusted predictors of AET discontinuation using Cox and Fine-and-Gray regressions

| **Predictors of AET discontinuation (unadjusted)** | Cause-specific Cox model |  | Fine-and-Gray subdistribution model |
| --- | --- | --- | --- |
|  | *CSHR^a^ [95% CI]* |  | *SDHR^b^ [95% CI]* |
| **Global cardiovascular adherence^c^** |  |  |  |
| Total adherence | **Ref.** |  | **Ref.** |
| Partial adherence | **1.26 [1.20; 1.32]***** |  | **1.23 [1.18; 1.29] ***** |
| Total non-adherence | **1.59 [1.50; 1.70]***** |  | **1.55 [1.45; 1.65] ***** |
| **Number of prescribed cardiovascular drug classes** |  |  |  |
| 2 | Ref. |  | Ref. |
| 3 | 1.09 [1.04; 1.15]*** |  | 1.09 [1.04; 1.14]*** |
| 4 | 1.15 [1.08; 1.24]*** |  | 1.14 [1.07; 1.22] *** |
| 5-6 | 1.28 [1.15; 1.43]*** |  | 1.25 [1.12; 1.40] *** |
| **Age at baseline** |  |  |  |
| ≤ 70 | Ref. |  | Ref. |
| 71-75 | 1.18 [1.11; 1.25]*** |  | 1.19 [1.12; 1.26]*** |
| 76-80 | 1.46 [1.37; 1.56]*** |  | 1.44 [1.35; 1.54] *** |
| > 80 | 1.92 [1.81; 2.03]*** |  | 1.84 [1.74; 1.95] *** |
| **AET drug switch** |  |  |  |
| 0 | Ref. |  | Ref. |
| 1 | 1.81 [1.70; 1.93]*** |  | 1.91 [1.79; 2.03]*** |
| ≥ 2 | 2.61 [2.42; 2.83]*** |  | 2.81 [2.58; 3.05]*** |
| **Type of BC surgery** |  |  |  |
| Tumorectomy | Ref. |  | Ref. |
| Total mastectomy | 1.14 [1.08; 1.20]*** |  | 1.07 [1.02; 1.13]** |
| **Time since initiation of any cardiovascular treatment** |  |  |  |
| < 5 years | Ref. |  | Ref. |
| ≥ 5 years | 0.59 [0.56; 0.63]*** |  | 0.59 [0.56; 0.62]*** |
| **Number of comorbidities (besides BC)** |  |  |  |
| 0 | Ref. |  | Ref. |
| 1 | 1.19 [1.13; 1.26]*** |  | 1.18 [1.12; 1.24]*** |
| ≥ 2 | 1.33 [1.26; 1.40]*** |  | 1.29 [1.22; 1.36]*** |
| **Number of hospital stays in the year before AET** |  |  |  |
| 0 | Ref. |  | Ref. |
| 1 | 1.11 [1.04; 1.18]*** |  | 1.06 [0.99; 1.12] |
| ≥ 2 | 1.23 [1.12; 1.35]*** |  | 1.12 [1.02; 1.23]* |
| **Adjuvant chemotherapy in the year before AET** |  |  |  |
| No | Ref. |  | Ref. |
| Yes | 0.85 [0.81; 0.90]*** |  | 0.71 [0.67; 0.75]*** |
| **FDEP^d^** |  |  |  |
| Q1 | 1.16 [1.09; 1.25]*** |  | 1.17 [1.10; 1.26]*** |
| Q2 | 1.10 [1.03; 1.18]** |  | 1.10 [1.03; 1.18]** |
| Q3 | 1.07 [1.00; 1.15]* |  | 1.09 [1.01; 1.16]* |
| Q4 | 1.02 [0.95; 1.09] |  | 1.03 [0.96; 1.10] |
| Q5 | Ref. |  | Ref. |
| **Financial aid for complementary health insurance** |  |  |  |
| No | Ref. |  | Ref. |
| Yes | 1.05 [0.94; 1.18] |  | 1.04 [0.92; 1.16] |

Abbreviations: *BC* breast cancer; *AET* adjuvant endocrine therapy; *CSHR* cause-specific hazard ratio; *SDHR* subdistribution hazard ratio

^a^ Cause-specific hazard ratios are interpreted as the adjusted effect of the covariate on the instantaneous rate of AET discontinuation among women currently event-free, that is, who have not discontinued AET, died or experienced cancer recurrence up to time t

^b^ Subdistribution hazard ratios are interpreted as the impact of a given covariate on the cumulative incidence of AET, that is, on the probability that AET discontinuation occurred in the population by time t

^c^ Patients were classified as fully adherence, partially adherent or fully non-adherent if they adhered to all, some or none of their cardiovascular drugs

^d^ The FDEP is a French index of social deprivation which summarises median household income, percentage of high school graduates in the population aged over 15, percentage of blue-collar workers in the active population and unemployment rate. Q1 is the least deprived quintile

Figures S1. Log-minus-log plots for each time-invariant predictor


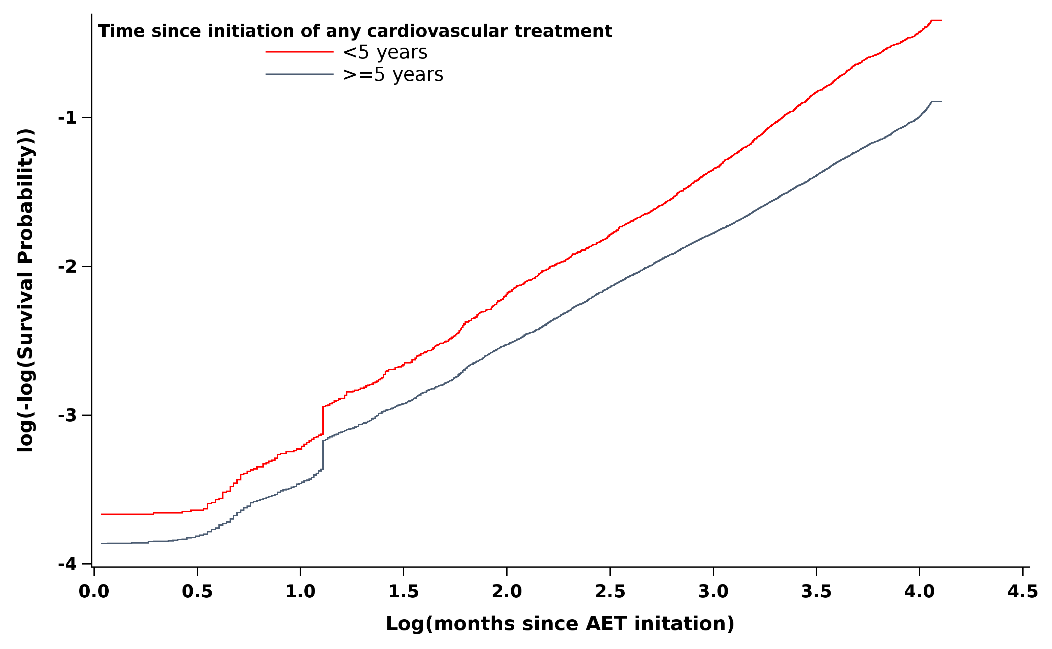

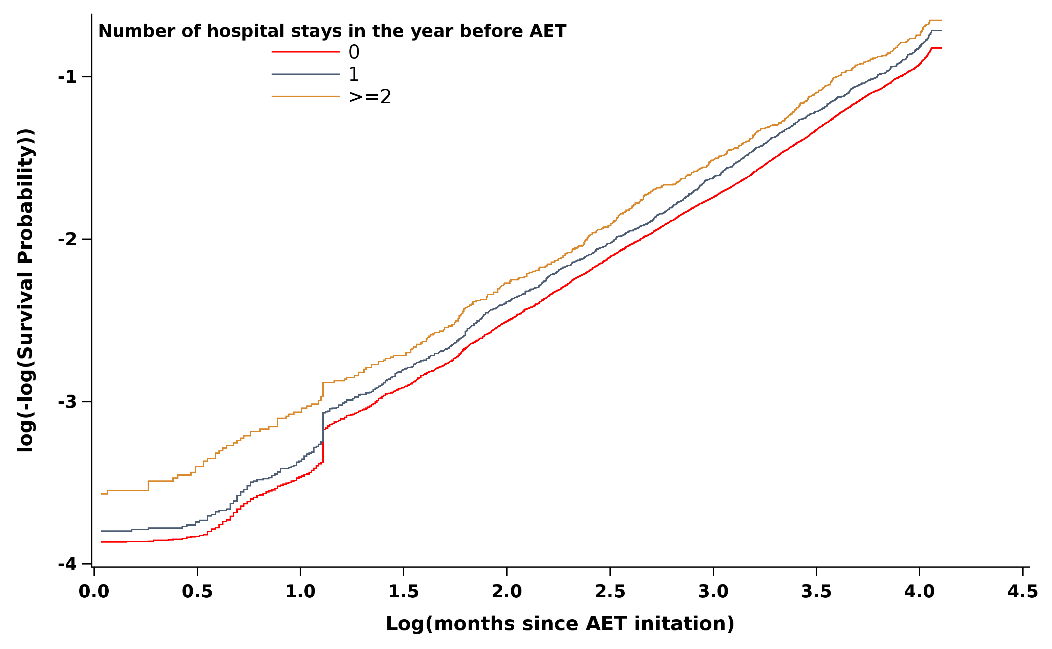

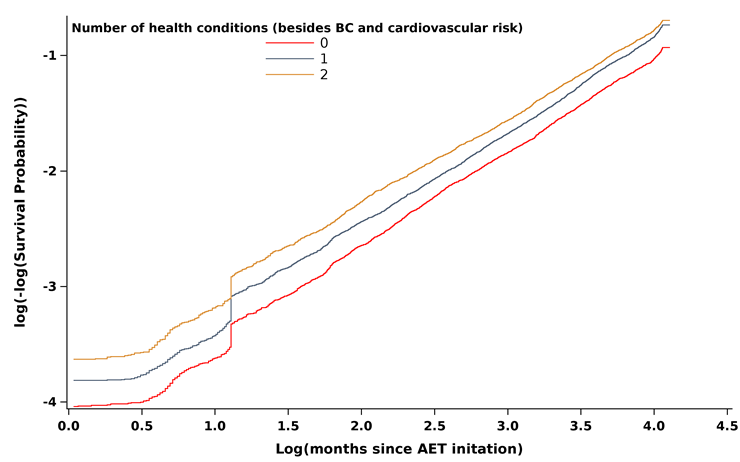

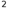

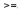

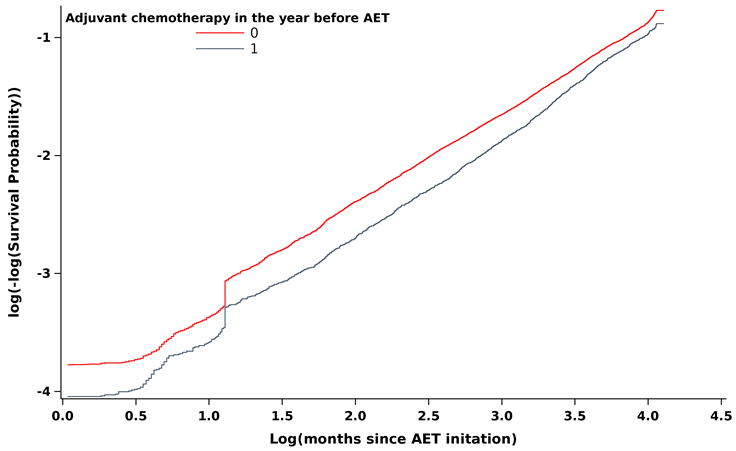

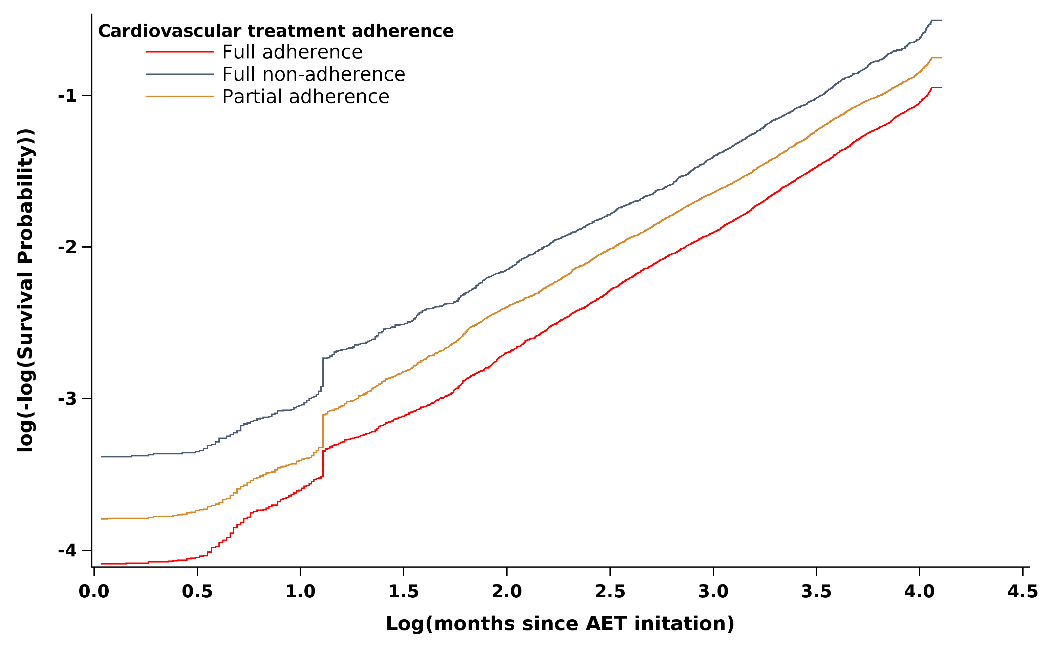

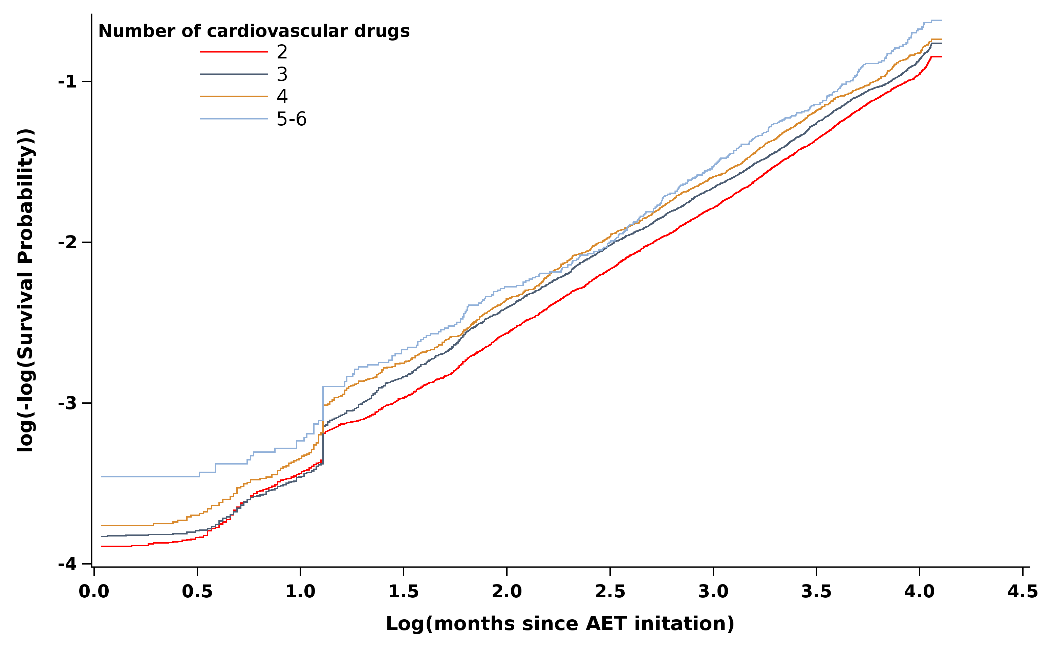

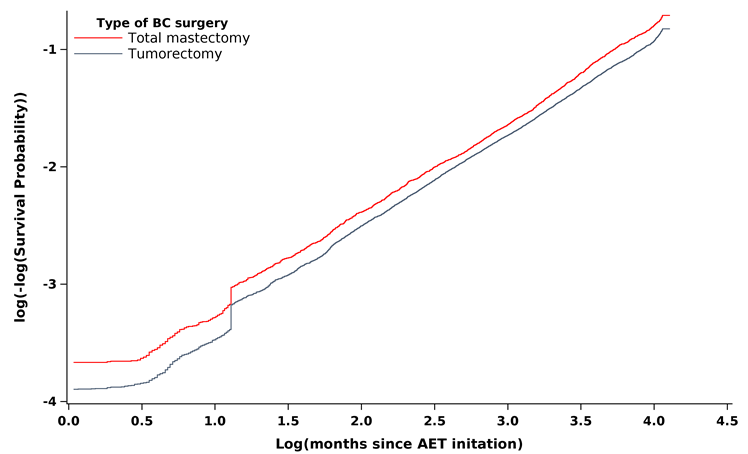

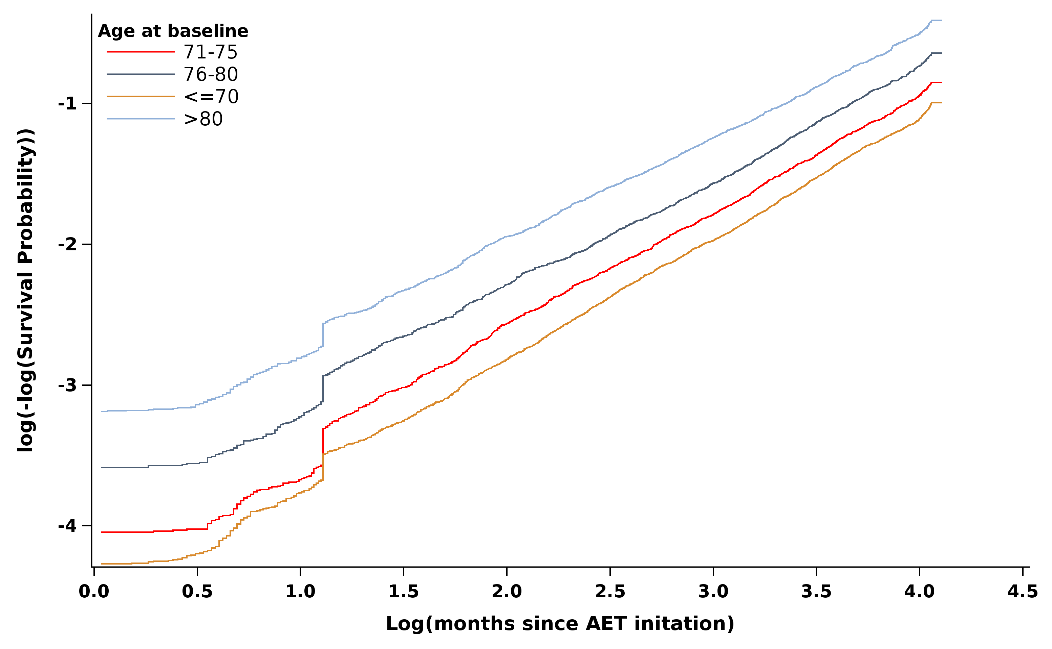


Abbreviations: *BC* Breast Cancer; *AET* adjuvant endocrine therapy


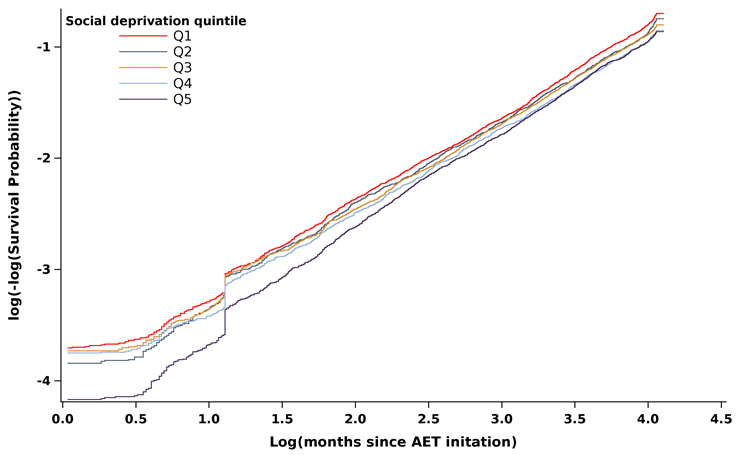

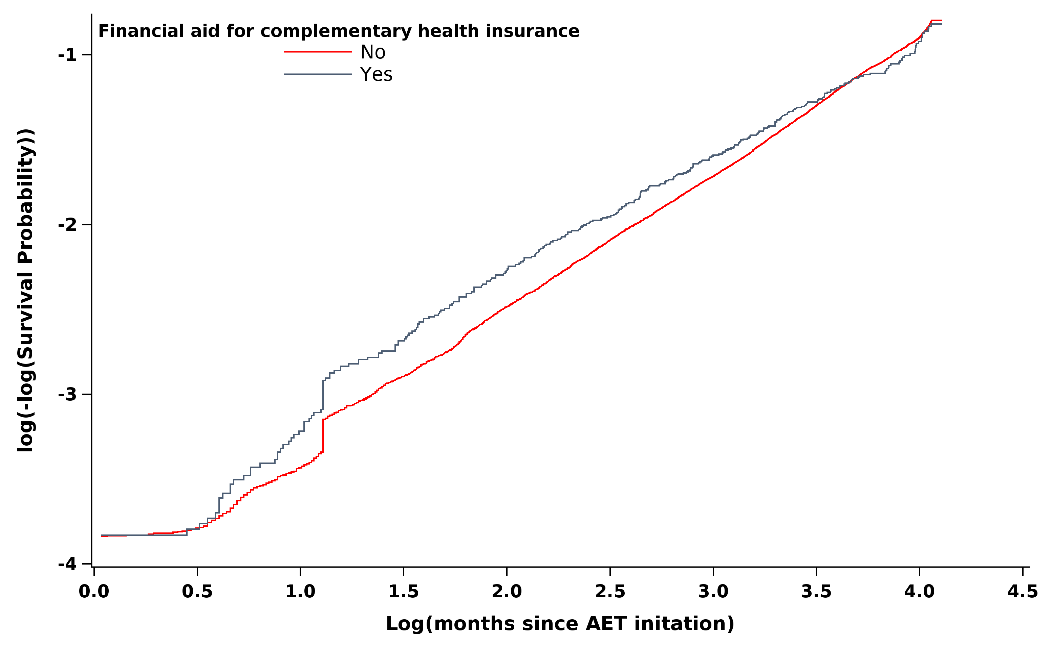

Supplement: Supplementary file 1 — Additional file 1: Tables S1. List of AET drugs with a dosing regimen of 2 pills per day. Table S2. List of health conditions considered for the comorbidity count included in multivariate models. Table S3. Population characteristics stratified by level of cardiovascular treatment adherence. Table S4. Unadjusted predictors of AET discontinuation using Cox and Fine-and-Gray regressions. Figures S1. Log-minus-log plots for each time-invariant predictor. [file 12916_2023_3156_MOESM1_ESM.docx]
